# Dynamic physiological temperature and pressure sensing with phase-resolved low-coherence interferometry

**DOI:** 10.1364/OE.27.005641

**Published:** 2019-02-14

**Authors:** J. M. Coote, E. J. Alles, S. Noimark, C. A. Mosse, C. D. Little, C. D. Loder, A. L. David, R. D. Rakhit, M. C. Finlay, A. E. Desjardins

**Affiliations:** 1 Department of Medical Physics and Biomedical Engineering, University College London, Gower Street, London WC1E 6BT, United Kingdom; 2 Wellcome/EPSRC Centre for Interventional and Surgical Sciences, University College London, Charles Bell House, 43-45 Foley Street, London W1W 7TS, United Kingdom; 3 Materials Chemistry Research Centre, Department of Chemistry, University College London, 20 Gordon Street, London WC1H 0AJ, United Kingdom; 4 The Royal Free Hospital, Pond Street, London NW3 2QG, United Kingdom; 5 Institute for Women’s Health, University College London, 86-96 Chenies Mews, London WC1E 6HX, United Kingdom; 6 Barts Heart Centre, St Bartholomew’s Hospital and Queen Mary University of London, Charterhouse Square, London EC1M 6BQ, United Kingdom

## Abstract

We report the development and characterisation of highly miniaturised fibre-optic sensors for simultaneous pressure and temperature measurement, and a compact interrogation system with a high sampling rate. The sensors, which have a maximum diameter of 250 µm, are based on multiple low-finesse optical cavities formed from polydimethylsiloxane (PDMS), positioned at the distal ends of optical fibres, and interrogated using phase-resolved low-coherence interferometry. At acquisition rates of 250 Hz, temperature and pressure changes of 0.0021 °C and 0.22 mmHg are detectable. An *in vivo* experiment demonstrated that the sensors had sufficient speed and sensitivity for monitoring dynamic physiological pressure waveforms. These sensors are ideally suited to various applications in minimally invasive surgery, where diminutive lateral dimensions, high sensitivity and low manufacturing complexities are particularly valuable.

## 1. Introduction

Many fields of clinical practice benefit from accurate, minimally invasive and localised measurements of pressure. Examples include measurements of pressure differences across coronary stenoses to assess their severity, monitoring of intracranial pressure changes following neurological interventions, and ureteric manometry [1]. Invasive temperature measurements are also of critical importance in many medical areas, including monitoring of ablation [2], cardiac output [3], and arterial metabolism [4]. These applications require highly miniaturised devices that can be readily integrated into catheters, guidewires and needles with lumens of less than 0.3 mm. Fibre-optic sensors that can detect multiple parameters with a single fibre and sensing element are well-suited to meet these requirements. Concurrent pressure and temperature measurements can also provide more valuable data than measurement of a single parameter alone, and independent temperature measurements can be used to compensate for errors in pressure measurement caused by cross-sensitivities.

Fibre-optic pressure and temperature sensors are commonly based on Fabry-Pérot (FP) cavities [5–26] and fibre Bragg gratings (FBG) [27–30]. FBGs can be coupled with FP cavities to produce combined temperature and pressure sensors [12,13,22]; other dual-parameter techniques include tapered and micro-structured fibres [31] and multiple optical cavities [14,24–26]. Many FP fibre optic pressure sensors have an inorganic (e.g. glass or silicon) membrane situated at the distal end that deforms with changes in external pressure, with the FP cavity formed between the deformable membrane and the distal end of the optical fibre [7–14]. With these sensors, fabrication techniques can be complex and result in costs that are incompatible with some single-use devices.

Polymer-based FP sensors are potentially advantageous as they can be fabricated with simple techniques and low-cost materials [15–26]. The Young’s moduli of polymers can be sufficiently small to allow for relatively thick membranes (e.g. 10 µm to 200 µm) in FP fibre optic pressure sensors, as compared with inorganic membranes (typically less than 5 µm in thickness). The high thermal expansion coefficients of some polymers are also advantageous for temperature measurements with high sensitivity. For example, optically transparent polymers have been used to form temperature-sensitive optical cavities [17,18,20]. Polydimethylsiloxane (PDMS) is of particular interest because of its biocompatibility, simple processing methods, optical transparency, and its high thermal expansion coefficient (900 × 10^−6^ °C^−1^ to 940 × 10^−6^ °C^−1^ [32]) and low Young’s modulus (0.36 MPa to 2.97 MPa [33,34]).

Several studies have investigated polymer-based optical cavities on fibre-optic sensors for simultaneous temperature and pressure sensing [24–26]. As yet, these devices have not been used for dynamic pressure measurements or tested in physiological conditions. Furthermore, studies focused on combined pressure-temperature probes have largely used low readout rate schemes, for instance those employing an optical spectrum analyser (OSA) and peak detection methods. High readout rates are essential for *in vivo* applications: The Association for the Advancement of Medical Instrumentation (AAMI) recommends 200 Hz for invasive blood pressure transducers [1].

In this study, we have developed a novel fibre-optic sensor design based on low-finesse optical cavities formed from PDMS, including a pressure-insensitive element for temperature measurement, and a flexible membrane for pressure measurement. These cavities are interrogated using low-coherence interferometry [35–37], thereby allowing for multiple optical path differences to be measured simultaneously with a single optical spectrum. We also present a console that provides high readout rates suitable for intravascular applications, and has a compact design. Below, we describe the construction and characterisation of these devices, and their use *in vivo*.

## 2. Sensor description

Figure 1(a)

**Fig. 1:**
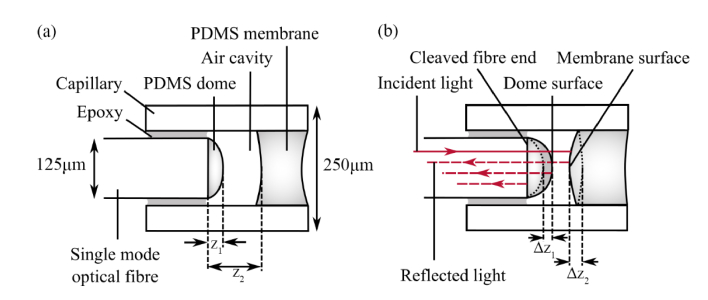
(a) Sensor element construction and geometry, showing the fibre-dome surface distance *z*_1_ and fibre-membrane inner surface distance *z*_2_. (b) Incident light is reflected from the cleaved fibre end, the dome outer surface and the membrane inner surface; it propagates back along the fibre, and the resulting spectral interference pattern (not shown) depends on the distances *z*_1_ and *z*_2_. The variation in the distance *z*_1_ depends only on temperature, and the variation in the distance *z*_2_ depends on both temperature and pressure, i.e. Δ*z*_1_(*P*,*T*) = Δ*z*_1_(*T*) and Δ*z*_2_(*P*,*T*) = Δ*z*_2_(*P*) + Δ*z*_2_(*T*).

shows a schematic diagram of the sensor element. Each sensor was made from a single mode optical fibre with a wavelength range of 830 nm to 980 nm and a cladding diameter of 125 µm (SM800-5.6-125, Thorlabs), cleaved at 90° to the optical axis. A droplet of PDMS (734, Dow Corning) was deposited on the cleaved fibre end, which assumed a dome shape (hereon referred to as a “dome”). A PDMS membrane was formed by drawing a bead of PDMS into the bore of a quartz capillary tube with an external diameter of 250 µm. The PDMS formed a plug with two concave surfaces which, after curing in air, acted as flexible membrane at the end of the capillary. Finally, the PDMS-tipped fibre was inserted into the capillary and fixed in position with epoxy, creating an air cavity inside the capillary.

### 2.1 Sensor mechanism

Each interface in the sensor element where a refractive index difference is present forms a reflective surface. Light from the fibre is partially reflected and partially transmitted at each interface, such that the interfaces form a set of low-finesse optical cavities. The sensor converts changes in pressure and temperature into changes in the lengths of these cavities as shown in Fig. 1(b): an increase in pressure in the medium surrounding the sensor causes the membrane to deform inwards towards the fibre and the distance denoted *z*_2_ in Fig. 1(a) decreases. As temperature increases, the dome expands and the distance denoted *z*_1_ increases; we observed that the dome is insensitive to pressure changes within the range of interest (760 mmHg to 1060 mmHg absolute). Therefore, the change in *z*_1_ as a function of pressure *P* and temperature *T*, denoted as Δ*z*_1_(*P*,*T*), can be written as Δ*z*_1_(*P*,*T*) = Δ*z*_1_(*T*). The membrane also undergoes thermal expansion; with an increase in temperature, the inner surface of the membrane moves towards the fibre, and vice-versa. Therefore, the change in *z*_2_ as a function of *P* and *T* can be written as: Δ*z*_2_(*P*,*T*) = Δ*z*_2_(*P*) + Δ*z*_2_(*T*).

We use the following method to measure pressure and temperature independently. First, the empirical relations Δ*z*_1_(*T*), Δ*z*_2_(*P*), and Δ*z*_2_(*T*) are determined by calibration, with one parameter held constant in each case. To measure pressure and temperature when neither is constant, we first measure the temperature using Δ*z*_1_(*T*) and then subtract the temperature-dependent component Δ*z*_2_(*T*) from Δ*z*_2_(*P*,*T*) to obtain Δ*z*_2_(*P*).

### 2.2 Interrogation method

**Fig. 2:**
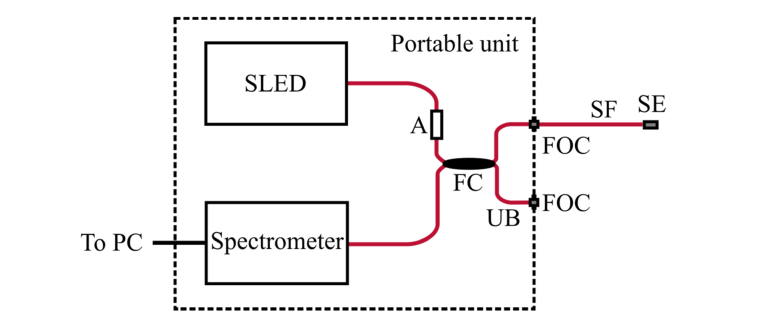
Schematic diagram of the optical sensor interrogation setup; SLED: superluminescent light emitting diode; A: attenuator; FC: 50:50 fibre-optic coupler; UB: unused branch; FOC: fibre-optic connector; SF: sensor fibre; SE: sensor element; PC: personal computer. The components integrated into the portable unit are shown enclosed by the dashed box.

The cavity length changes Δ*z*_1_ and Δ*z*_2_ were measured using phase-resolved low coherence interferometry (LCI) with a self-referenced fibre-optic Michelson interferometer. A superluminescent light emitting diode (SLED) with a central wavelength of 830 nm, a spectral width of 65 nm and an output power of 15 mW (BLM-S-820-B-I-10, Superlum) was connected to one input branch of a 50:50 fibre-optic coupler (TW850R5A2, Thorlabs). The second input branch was connected to a compact broadband spectrometer (Flame-S, Ocean Optics), with an acquisition time of 1 ms. The fibre-optic sensor under test was connected to one of the output branches of the coupler, and the second output branch was unused in this study; it could, however, be used for concurrent interrogation of two sensors. To prevent saturation of the spectrometer detector, an in-line attenuator (VOA-850-APC, Thorlabs) was placed between the SLED and the coupler. Raw spectra were acquired and processed by a personal computer running a custom program written with LabVIEW (National Instruments), with an overall sampling rate of up to 250 Hz. The interrogation components were integrated into a portable unit with dimensions of 30 cm × 20 cm × 9 cm. A schematic representation of this console unit is shown in Fig. 2

.

Light reflected by the interfaces inside the sensor element follows a common path from the sensor to the spectrometer, and results in interference fringes in the detected spectrum. The absolute geometric distance between two interfaces (measured along the optical axis) is denoted as *z* = *z*_0_ + Δ*z*, where *z*_0_ is the baseline distance and Δ*z* is a small variation in *z* that is caused by pressure and temperature changes. If the medium between the interfaces has a constant refractive index *n*, the optical path difference between the waves reflected from these interfaces, denoted z′, is given by: z′ = z_0_′ + Δz′, where *z*_0_′ = 2*nz*_0_ and Δ*z*′ = 2*n*Δ*z*. Considering only two reflecting surfaces, for instance the cleaved distal end of the optical fibre and the outer surface of the dome, the intensity of the received spectrum as a function of wavenumber *k* (where *k* = 1/*λ* and *λ* is the wavelength) is given by [39]:

$$I(k)=S(k)[R_{1}+R_{2}+2\sqrt{R_{1}R_{2}}\cos(2\pi kz\prime )],$$ (1)

where *S*(*k*) is the intensity spectrum of the SLED, and *R*_1_ and *R*_2_ are the intensity reflection coefficients of the first and second surface respectively. The wavenumber *k* can be expressed as *k* = *k*_0_ *+* Δ*k*, where *k*_0_ is the central wavenumber of the source. Assuming that Δ*z* and Δ*k* are small compared to *z*_0_ and *k*_0_, the cosine argument in Eq. (1) can be approximated by neglecting a second-order term, as follows:

$$kz\prime =k(z_{0}^{\prime }+\Delta z\prime )=kz_{0}^{\prime }+k_{0}\Delta z\prime +\Delta k\Delta z\prime \approx kz_{0}^{\prime }+k_{0}\Delta z\prime .$$ (2)

Due to the low numerical aperture of the fibre (0.10 to 0.14) and the low reflectance of the interfaces, we assume two-beam interference, with normal incidence. Dependencies of *I*(*k*) on the polarisation state of light incident on the sensor element are also neglected, since this dependency was observed to be negligible (see Section 3.4).

Combining Eqs. (1) and (2) and taking the inverse Fourier transform, we obtain:

$$\begin{array}{l} {ℑ}^{-1}[I(k)](z\prime )={ℑ}^{-1}[S(k)](z\prime )*\sqrt{2\pi }\{(R_{1}+R_{2})\delta (z\prime )\\ \;+\sqrt{R_{1}R_{2}}[\delta (z\prime -z_{0}^{\prime })\exp\left(i2\pi k_{0}\Delta z\prime \right)+\delta \left(z\prime +z_{0}^{\prime }\right)\exp\left(-i2\pi k_{0}\Delta z\prime \right)]\}, \end{array}$$ (3)

where * represents convolution. The first term in the product of the right hand side of Eq. (3) is the inverse Fourier transform of the source spectrum, which is centred around *z′* = 0. The second term provides information about the periodicity of the interference pattern, which depends on the optical path difference *z*_0_*′*; therefore, *z*_0_*′* can be found by locating the maxima of the magnitude of the inverse Fourier transform shown in Eq. (3), which occur at *z′* = ± *z*_0_*′*. This absolute optical path difference is only measurable to a precision determined by the resolution of the inverse Fourier transform, which depends on the wavelength range of the source spectrum. However, small variations in the optical path difference, Δ*z′*(*t* - *t*_0_), can be monitored at much higher resolution using the complex argument of Eq. (3), taking *z′* = + *z*_0_*′*:

$$\phi (t-t_{0})\equiv \arg\{{ℑ}^{-1}[I(k)](+z_{0}^{\prime })\}=2\pi k_{0}\Delta z\prime (t-t_{0}),$$ (4)

where the time *t* is referenced to an arbitrary starting point *t*_0_. It is seen that *ϕ*(*t - t*_0_) is proportional to the change in cavity length Δ*z*(*t - t*_0_), and therefore it is also proportional to the pressure *P*(*t* – *t*_0_) and the temperature *T*(*t* – *t*_0_), assuming that *n* is constant with time [35,36]. For brevity, in what follows, we have adopted the notation *ϕ* to indicate *ϕ*(*t - t*_0_).

When more than two reflecting surfaces are present, each pair of reflectors generates a distinct maximum in the inverse Fourier-transformed spectrum, located at $z\prime =z\prime_{j}=2nz_{j}$, where *j* = 1,2,… denotes the *j*^th^ peak. Using Eq. (4), the complex argument *ϕ_j_* can be obtained for each maximum, thereby allowing multiple cavity lengths to be measured simultaneously with the same spectrum acquisition.

**Fig. 3:**
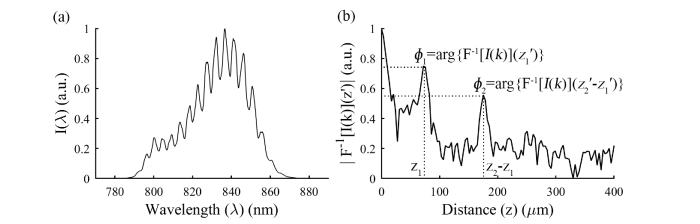
Examples of the raw and processed signals: (a) raw intensity spectrum versus wavelength; the spectrum is resampled so that it is linear in wavenumber prior to inverse Fourier transformation; (b) magnitude of the inverse Fourier-transformed spectrum (on a logarithmic scale) with peaks corresponding to *z*_1_ and *z*_2_ – *z*_1_ labelled; *z′* has been converted to *z* by taking *n* = 1; *ϕ*_1_ and *ϕ*_2_ are obtained using the complex argument of the inverse Fourier-transformed spectrum at distance axis locations *z*_1_′ and *z*_2_′-*z*_1_′ .

Examples of a raw spectrum acquired from a sensor, and its inverse Fourier-transformed spectrum, are shown in Fig. 3

. In the inverse Fourier-transformed spectrum (Fig. 3(b)), two maxima are visible. The locations on the distance axis of these maxima correspond to the fibre-dome distance (*z*_1_ in Fig. 1), and the dome-membrane distance (*z*_2_ - *z*_1_ in Fig. 1). According to Eq. (4), we can obtain two differential signals, referred to as *ϕ*_1_ and *ϕ*_2_, by taking the complex arguments of the inverse Fourier-transformed spectrum at these two locations.

The pressure and temperature dependence of these signals may be expressed as:

$$\left[\begin{array}{c} \phi_{1}\\ \phi_{2} \end{array}\right]=\left[\begin{array}{cc} m_{1T} & m_{1P}\\ m_{2T} & m_{2P} \end{array}\right]\left[\begin{array}{c} T(t-t_{0})\\ P(t-t_{0}) \end{array}\right],$$ (5)

where *T*(*t – t_0_*) and *P*(*t – t_0_*) are the changes in temperature and pressure since the beginning of the measurement (*t* = *t_0_*). The coefficients *m_jT_* and *m_jP_* (*j* = 1,2) are the sensitivities of the sensor signals *ϕ_j_* to temperature and pressure respectively, and are established by calibration (see Section 3). We can then recover temperature and pressure changes from the sensor signals by inverting the square matrix in Eq. (5). To perform absolute (rather than differential) temperature and pressure measurements, an initial reading must be taken both from the sensor and from independent reference temperature and pressure sensors to establish the initial readings of *ϕ*_1_ and *ϕ*_2_ and initial values for temperature and pressure at time *t* = *t*_0_.

The smallest measurable temperature and pressure changes are determined by the noise in the optical system, which limits the resolution of the signals *ϕ*_1_ and *ϕ*_2_. In the shot-noise limited case, this resolution depends on the signal to noise ratio (SNR) of the interferometric measurement, according to Park et al. [37]:

$$\sigma_{\phi j}={\left(SNR\right)}^{-1/2},$$ (6)

where *σ_ϕj_* is the standard deviation of the measured signal *ϕ_j_*. The SNR (in decibels) is given by [38]:

$$SNR=20\;\log\left(\frac{〈{ℑ}^{-1}[I(k)](z_{j}^{\prime })〉}{\sigma_{bg}}\right),$$ (7)

where $〈{ℑ}^{-1}[I(k)](z_{j}^{\prime })〉$ is the mean of the complex modulus of the Fourier-transformed spectrum at the *j*^th^ peak, and *σ_bg_* is the standard deviation of the background signal at the same depth position as the peak, when no reflector is present. Using Eq. (7) and the reflected intensity spectrum obtained from Sensor 3 (see next section) we calculated the SNRs for *ϕ*_1_ and *ϕ*_2_ as 54 dB and 43 dB, giving standard deviations of *σ_ϕ_*_1_ = 0.0021 rad and *σ_ϕ_*_2_ = 0.0068 rad, respectively.

In practice, since the spectra provided by the spectrometer are expressed in terms of intensity versus wavelength, the sampling points of the spectra are no longer uniformly spaced when converted into wavenumbers; therefore, the spectra are linearly interpolated on to a uniformly spaced wavenumber axis to allow an inverse fast Fourier transform (IFFT) algorithm to be applied. Additionally, since the complex argument function is only single-valued in the range –π < *ϕ* < π, the sensor signals *ϕ_j_* have discontinuities at intervals of 2π; therefore, a phase-unwrapping algorithm is applied to *ϕ_j_* to obtain continuous signals.

## 3. Sensor characterisation

Sensors were characterised inside a sealed water-filled tube immersed in a water bath. The pressure was regulated by an electropneumatic regulator (SMC Pneumatics), and monitored by a commercial reference pressure transducer with a calibrated range of 750 mmHg to 1500 mmHg (absolute) and an accuracy of ± 0.08% (Omega Engineering). An exposed junction K-type thermocouple with a nominal accuracy of ± 0.25% (RS Components) was positioned in the tube next to the sensor under test to provide reference temperature readings.

### 3.1 Pressure and temperature sensitivity, resolution and uncertainty

Here, sensitivity was defined as the change in the sensor phase signal *ϕ_j_* that results from a known change in temperature or pressure. To characterise the sensors’ pressure and temperature sensitivities, sensor signals were recorded as either the pressure or temperature was increased and the other parameter was held constant. Pressure sensitivities were tested over the range 760 mmHg to 1060 mmHg absolute (0 mmHg to 300 mmHg gauge; these values are provided as gauge pressure is typically used in clinical practice) at 20 °C; temperature sensitivities were tested over the range 20 °C to 40 °C at 760 mmHg absolute (0 mmHg gauge). These ranges were chosen to be relevant to physiological environments. The readings of *ϕ*_1_ and *ϕ*_2_ were then plotted against the reference sensor readings to obtain calibration plots, and least-squares best-fit lines were fitted to the data. The gradients of these best-fit lines indicated the pressure and temperature sensitivities of the dome and membrane, i.e. *m_jT_* and *m_jP_* (*j* = 1,2) in Eq. (5). In all the sensors tested, *ϕ*_1_ showed no observable pressure sensitivity, i.e. *m*_1_*_P_* = 0 rad/mmHg. All other calibration plots obtained were linear, with R^2^ values greater than 0.98. Three sensors were calibrated, and their sensitivities are shown in Table 1

**Table 1:** Sensor characteristics

Parameter	Sensor 1	Sensor 2	Sensor 3
m_1T_ (rad/°C)	0.7094 ± 0.0022	0.2850 ± 0.0013	0.272443 ± 9.4 × 10^−5^
m_2T_ (rad/°C)	−3.1288 ± 0.0092	−3.123 ± 0.016	−1.64525 ± 8.7 × 10^−4^
m_2P_ (rad/mmHg)	−0.040621 ± 2.0 × 10^−5^	−0.037761 ± 1.2 × 10^−5^	−0.059145 ± 5.8 × 10^−5^
ΔT_min_ (°C)	0.0021	0.012	0.0076
ΔP_min_ (mmHg)	0.64	0.40	0.22
Uncertainty (ΔT) (%)^a^	± 0.31	± 0.46	± 0.051
Uncertainty (ΔP) (%)^a^	± 0.70	± 0.46	± 0.48

^a^ Percentage of full scale: Δ*T*_max_ = 40°C; Δ*P*_max_ = 300 mmHg

, along with uncertainties indicating 95% confidence bounds.

We defined the resolution of the sensors as the minimum detectable change in temperature and pressure, which depended on the size of random fluctuations in the signals due to various noise sources and small environmental disturbances. To estimate the magnitudes of these fluctuations, the sensors were placed in the characterisation setup, and signals were recorded at room temperature and atmospheric pressure. The standard deviations of *ϕ*_1_ and *ϕ*_2_ were calculated using 50 consecutive samples of this data across a period of approximately 0.5 s, in which time the fluctuations in ambient temperature and pressure were assumed to be negligible. The standard deviations of *ϕ*_1_ and *ϕ*_2_, denoted *σ_ϕ_*_1_ and *σ_ϕ_*_2_, were converted to temperature and pressure resolutions by dividing *σ_ϕ_*_1_ by the temperature sensitivity *m*_1_*_T_*, and *σ_ϕ_*_2_ by the pressure sensitivity *m*_2_*_P_*, to obtain the temperature and pressure resolutions, Δ*T*_min_ and Δ*P*_min_ for each sensor.

Finally, to estimate the uncertainty in the pressure and temperature measurements recovered using Eq. (5), the uncertainties in the calculated sensitivities *m_jT_* and *m_jP_* were combined with the standard deviations of *ϕ*_1_ and *ϕ*_2_, using 95% confidence bounds and the standard formula for propagation of errors [40]. All sensor characteristics described above are summarised in Table 1.

### 3.2 Simultaneous pressure and temperature measurement

To demonstrate simultaneous pressure and temperature measurement, a sensor was placed inside the characterisation setup described above, and pressure was cycled between 760 mmHg and 860 mmHg absolute with a hold time of 1 s at each pressure; this range was chosen to simulate intracoronary pressure in the cardiac cycle. At the same time, water at different temperatures between 20 °C and 40 °C was added to the water bath, which was stirred with a magnetic stirrer, to produce variations in temperature.

In a second experiment, a sensor was placed directly into the stirred water bath at ambient pressure, with the reference thermocouple and pressure transducer immersed alongside the sensor to the same water depth. Hot water was added to the water bath over a period of approximately 36 s to produce a temperature ramp; cold water was then added in stages over a period of 76 s to produce three descending temperature steps.

To calibrate the sensors for absolute temperature and pressure measurement, the initial readings from the sensor under test and the reference sensors were used to determine *ϕ*_1_, *ϕ*_2_, *T* and *P* at time *t* = *t*_0_. Then Eq. (5) was used to convert the sensor signals into changes in temperature and pressure over the course of the experiment.

Figure 4(a)

**Fig. 4:**
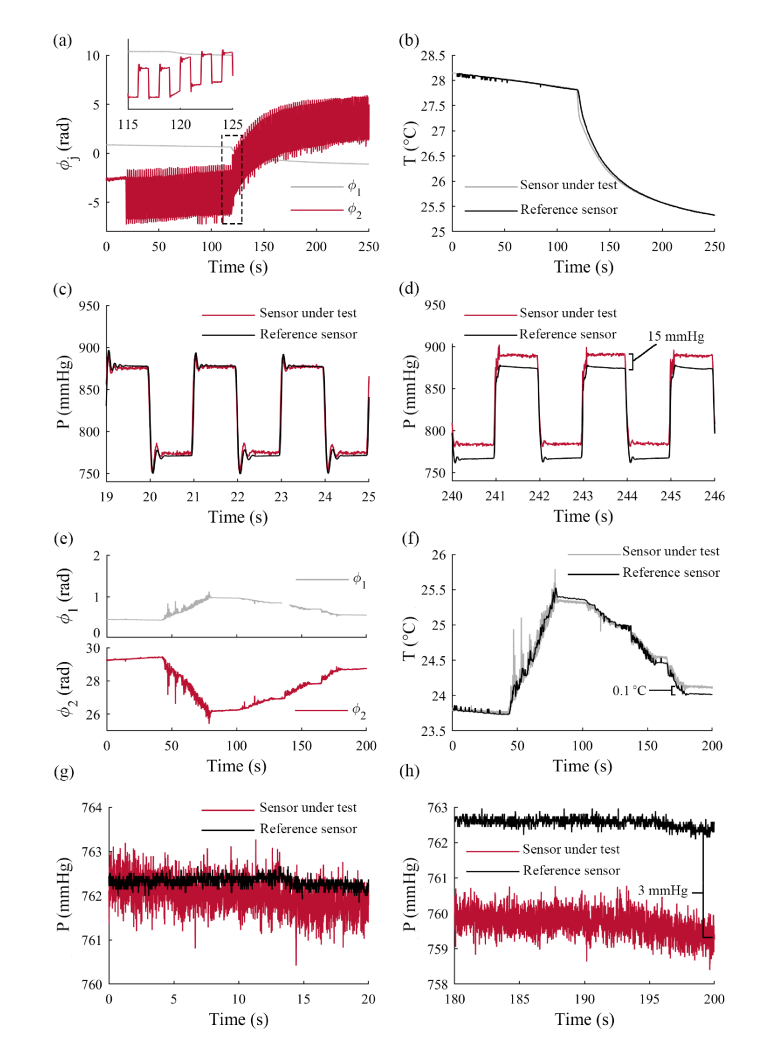
(a) – (d): Simultaneous pressure and temperature measurement during pressure cycling with a temperature change; (a) sensor signals as acquired; the inset shows an enlarged view of the region indicated by the dashed box; (b) calibrated temperature measurements and reference thermocouple measurements; (c) calibrated pressure measurements and reference pressure transducer measurements; (d) calibrated pressure measurements and reference pressure transducer measurements at a later time, showing a discrepancy of approximately 15 mmHg between the calibrated pressure measurements and reference pressure transducer measurements due to drift. (e) – (h): Simultaneous pressure and temperature measurements with a temperature ramp at constant pressure; (e) acquired sensor signals: *ϕ*_1_ (upper subplot) and *ϕ*_2_ (lower subplot); (f) calibrated temperature measurements and reference thermocouple measurements; (g) calibrated pressure measurements and reference pressure transducer measurements; (h) calibrated pressure measurements and reference pressure transducer measurements at a later time, showing a discrepancy of approximately 3 mmHg due to drift.

shows data acquired from the sensor during pressure cycling with a simultaneous temperature drop. The calibrated pressure and temperature readings are shown alongside the reference sensor data in Figs. 4(b)-4(d). The temperatures measured by the sensor agreed well with the reference sensor data (Fig. 4(b)). Initially, the pressures measured by the sensor also agreed well with the reference sensor data (Fig. 4(c)). However, as shown in Fig. 4(d), at a later time in the experiment (*t* = 240 s) the sensor signal had drifted by approximately 15 mmHg between the pressures recorded by the sensor under test and the reference pressure transducer.

Figure 4(e) shows data acquired from the sensor during the temperature ramp-up and step-down at ambient pressure, and the calibrated temperature and pressure measurements are shown alongside the reference sensor data in Figs. 4(f)-4(h). Again, the temperature measurements of the sensor and reference thermocouple agree well, with a discrepancy of 0.1 °C by the end of the experiment (Fig. 4(f)). The pressure measurements of the sensor and the reference pressure measurements also agree well initially, but again the sensor signal has drifted by 3 mmHg after 200 s, as shown in Fig. 4(h). The maximum discrepancy observed between the sensor data and reference transducer data was 4.7 mmHg.

### 3.3 Speed of response

The time constants for the temperature responses of the dome and membrane were measured by rapidly dipping the sensor element of Sensor 3 into a beaker of water at a constant temperature. The sensor was mounted on a vertical translation stage so that the sensor element was suspended in air above a beaker of water. The air temperature and water temperature were measured as 16 °C and 23 °C respectively. Signals from the sensor were recorded as the sensor was moved into the water using the translation stage. The data for *ϕ*_1_ and *ϕ*_2_ versus time *t* were then fitted to exponential functions of the form *ϕ* = *a* + *b*(1-exp(-*t*/*τ*)), where *τ* is the time constant. These time constants for the dome and membrane were found to be 1.9 s and 1.4 s respectively.

Time constants for the pressure response of the membrane were measured by placing Sensor 3 inside the characterisation setup, with the water at ambient temperature. Sensor signals were recorded as the pressure was stepped from 762 mmHg to 794 mmHg, and the data were fitted to an exponential function as described above. The pressure response time constant of the membrane signal was found to be 25 ms.

### 3.4 Polarisation sensitivity

When used *in-vivo,* the fibre-optic sensor will be subjected to bending and strain which may alter the birefringence of the fibre, causing a shift in the polarisation state of the light propagating inside the fibre. To investigate whether polarisation changes affect the sensor signals, a manual fibre polarisation controller (FPC030, Thorlabs) installed with bare single-mode fibre (780HP, Thorlabs) was connected between the SLED and the fibre-optic coupler. Sensors were placed inside the setup described above, at room temperature and atmospheric pressure, and signals were recorded while the polarisation controller paddles were rotated; these rotations created stress-induced birefringence in the fibre. The sensor signals *ϕ*_1_ and *ϕ*_2_ showed no observable changes in response to the induced changes to the polarisation state of the incident light, confirming that polarisation had a negligible effect on the sensor signals; these findings are consistent with the radial symmetry of the sensor element about the optical axis.

## 4. *In vivo* study

Sensors were integrated into catheters for delivery into blood vessels. Each catheter was made from fine bore polythene tubing (Portex), sealed at the distal end. The sensor was placed inside the tube, and a small hole in the side of the tube above the sensing element allowed fluid pressure into the catheter. A haemostatic valve with a side arm at the proximal end of the catheter allowed flushing of the catheter with saline.

**Fig. 5:**
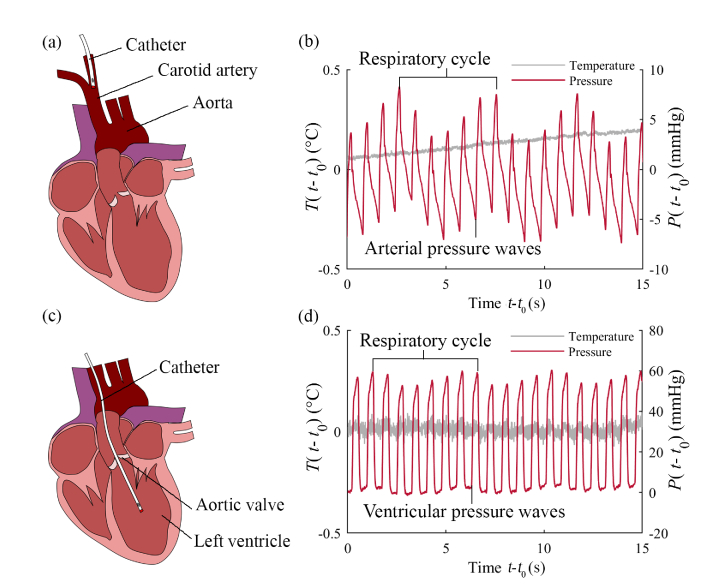
*In vivo* study: (a) Diagram of the ewe heart showing approximate position of the catheter in the right carotid artery; (b) relative temperature and pressure measurements (referenced to start time *t*_0_) acquired in real time in the carotid artery, with the pressure signal varying in response to arterial pressure waves with modulation due to respiration. The temperature signal is unaffected by pressure changes but gradually increases over time; (c) diagram showing approximate position of the catheter in the left ventricle; (d) relative temperature and pressure measurements acquired in real time from the left ventricle, with the temperature signal unchanging and the pressure signal responding to ventricular pressure, with modulation due to respiration.

*In vivo* work was performed in sheep at the Biological Services Unit, Royal Veterinary College, London, UK, under project license 70/7408 and PIL IAC41E0F9. All procedures on animals were conducted in accordance with U.K. Home Office regulations and the Guidance for the Operation of Animals (Scientific Procedures) Act (1986). Ethics approval was provided by the joint animal studies committee of the Royal Veterinary College and the University College London, United Kingdom. Experiments were performed in a single ewe under terminal anaesthesia. After clipping the wool and cleansing the skin over the neck with povidone iodine, the catheter was placed via an introducer sheath into the right carotid artery (Fig. 5(a)

) under ultrasound guidance. The catheter was advanced towards the heart for 5 cm and sensor signals were recorded in real time.

The recorded data were converted into pressure and temperature measurements using Eq. (5), and are shown in Fig. 5(b). Since no reference temperature or pressure probes were available during this experiment to determine the initial offsets of the sensor signals, the *in-vivo* data are presented as relative temperature and pressure changes. The pressure signal showed detailed waveforms in response to the pressure waves inside the artery, and an additional modulation with a period of approximately 5 s, which is thought to be due to respiration. The temperature-dependent signal showed no variation in response to pressure waves as expected, but a gradual upward drift of approximately 0.01 °C/s was observed, which was of unknown origin.

In a second measurement, the catheter was introduced into the right carotid artery and advanced into the left ventricle via the aorta and aortic valve (Fig. 5(c)). Sensor signals were recorded in real time and converted into relative temperature and pressure units (Fig. 5(d)). The pressure signal showed distinctive ventricular pressure waveforms, while the temperature-dependent signal was constant in time, varying by less than 0.003 °C/s. This measurement did not appear to be affected by drift.

## 5. Discussion

This study has shown that polymer-based fibre-optic sensors are capable of performing simultaneous measurements of dynamic pressure and temperature under physiological conditions. The sensor design employs the advantageous properties of PDMS such as high thermal expansion coefficient, low Young’s modulus and simple processing methods to achieve high pressure and temperature resolution with simple construction and low-cost materials.

The high sampling rate of the interrogation system (250 Hz) combined with the sensitivity of the sensors allows accurate measurement of rapidly changing dynamic variables, such as physiological pressure waveforms, as demonstrated by the *in vivo* results. This is in contrast to interrogation systems employing optical spectrum analysers (OSAs) and signal processing based on peak detection and tracking, where achieving high enough sampling rates for dynamic physiological measurement is challenging. The interrogation system is also compact and portable, making it well-suited for use in clinical settings.

The sensors have a maximum diameter of 250 µm, making them highly suitable for integration into catheters and guidewires for minimally invasive procedures, and further miniaturisation will be possible through the use of fibres and capillaries with smaller diameters.

The ability to acquire both temperature and pressure readings simultaneously, using a single sensing element, is also very beneficial from the perspective of device miniaturisation, and the potential for functionalisation of PDMS with nanoparticles, wavelength selective coatings, biological detection elements and nanostructures opens up the possibility of adding more sensing capabilities [41] and combining sensing with imaging [42] in a single probe.

The uncertainty and resolution of our sensors are comparable to those of some commercially available fibre-optic invasive pressure instruments [43,44]. However, our results suggest some routes to optimisation: for a given level of system and environmental noise, higher sensitivities will improve the resolution of the sensors. Temperature sensitivity can be increased by creating thicker PDMS domes, as linear thermal expansion is proportional to thickness. Pressure sensitivity can be improved by creating thinner PDMS membranes, or membranes with a lower Young’s modulus, that will undergo larger deformations under pressure. Thinner polymer membranes have the additional advantage of reduced temperature sensitivity, leading to lower uncertainty in the pressure measurements.

The measured temperature resolutions of our sensors are close to the theoretical limit predicted by Eq. (6): for Sensor 3, *σ_ϕ_*_1_ = 0.0021 rad, giving a theoretical temperature resolution of 0.0076 °C, which is the same as the measured temperature resolution (see Table 1). The measured pressure resolutions are lower than the theoretical limit (0.22 mmHg and 0.11 mmHg respectively, for Sensor 3). This difference may be due to the high temperature sensitivity of the membrane, which results in larger fluctuations in the membrane signal compared to the dome signal, in response to small environmental disturbances such as convection currents in the fluid. The membrane is also in direct contact with the surrounding medium, which may result in larger drift in the pressure-sensitive membrane signal due to absorption, desorption and diffusion of molecules between the membrane, air cavity and surrounding medium.

The long-term drift shown in Fig. 4(d) has been observed in all sensors tested, and we are currently investigating its causes and approaches to reducing or eliminating it. Drift has been observed in other studies on polymer-based fibre-optic pressure sensors [16], and has been attributed to optical heating of the Fabry-Pérot cavity by the interrogation light source, and absorption of water into the polymer. Our preliminary results suggest that optical heating in our sensor elements is negligible, but suggest that absorption, desorption and diffusion of molecules between the PDMS membrane, the air cavity inside the capillary and the medium surrounding the sensor are contributing factors. PDMS is known to be permeable to water vapour and atmospheric gases [33,45,46]. Improved stability might be achieved by using polymer materials with lower water absorption and diffusion properties, or by making use of water and gas-impermeable coatings; for example, diffusion of water could be reduced by incorporating a layer of Parylene C within the membrane [46].

To summarise, when combined with phase-resolved LCI, PDMS-based fibre-optic sensors are promising for a wide range of minimally invasive clinical applications. Future work will focus on further miniaturisation, exploiting the wide range of mechanical and chemical properties offered by polymer materials to optimise the sensors for stability and sensitivity, and including additional sensing and imaging capabilities.

## 6. Conclusions

We have developed all-optical pressure and temperature sensors based on deformable low-finesse optical cavities formed from PDMS, and a compact interrogation system with simple instrumentation and high-speed acquisition rates of up to 250 Hz. Pressure and temperature can be measured independently, using the different responses of the optical cavity lengths to temperature and pressure. *In vivo* experiments indicate that the sensors are sufficiently fast and sensitive to resolve physiological waveforms. The advantages of our approach include sensors with simple construction methods, low cost materials and the ability to acquire multiple parameters with a single, highly miniaturised device, and an interrogation system that provides acquisition speeds high enough to accurately monitor dynamic physiological parameters. These sensors are highly suitable for use in minimally invasive surgical procedures.
